# Combination of Four Serum Exosomal MiRNAs as Novel Diagnostic Biomarkers for Early-Stage Gastric Cancer

**DOI:** 10.3389/fgene.2020.00237

**Published:** 2020-03-17

**Authors:** Shuli Tang, Jianan Cheng, Yuanfei Yao, Changjie Lou, Liang Wang, Xiaoyi Huang, Yanqiao Zhang

**Affiliations:** ^1^Department of Gastrointestinal Medical Oncology, Harbin Medical University Cancer Hospital, Harbin, China; ^2^Translational Medicine Research and Cooperation Center of Northern China, Heilongjiang Academy of Medical Sciences, Harbin, China; ^3^Department of Tumor Biology, H. Lee Moffitt Cancer Center, Tampa, FL, United States; ^4^Biotherapy Center, Harbin Medical University Cancer Hospital, Harbin, China

**Keywords:** exosomes, microRNAs, gastric cancer, early diagnosis, liquid biopsy

## Abstract

Gastric cancer (GC) remains a leading cause of cancer-related mortality in the United States and China, there is an urgent need to discover novel non-invasive biomarkers for the early diagnosis of GC to improve the prognosis of GC patients. Exosomal miRNAs are considered promising biomarkers for cancer diagnosis. Using next-generation sequencing (NGS), bioinformatics and further validation, we identified and evaluated exosomal miRNAs in serum as early diagnostic markers for GC. NGS revealed that the average mappable reads in the RNA libraries were about 6.5 million per patient including miRNAs (73.38%), rRNAs (17.10%), snRNAs (8.83%), snoRNAs (0.65%), and tRNAs (0.04%). A total of 66 up and 13 down-regulated exosomal miRNAs were found in the screened cohort. In the validation cohort, by comparing with healthy individuals, higher levels of serum exosomal miR-92b-3p, let-7g-5p, miR-146b-5p, and miR-9-5p were found to be significantly associated with early-stage GC (*p* < 0.05). Diagnostic power of the combined panels of the exosomal miRNAs or the combination of exosomal miRNAs and CEA outperformed that of single exosomal miRNA marker for establishing a diagnosis of early-stage GC. The combined diagnosis of exosomal miR-92b-3p + let-7g-5p + miR-146b-5p + miR-9-5p with CEA had the most powerful efficiency with an AUC up to 0.786. In addition, serum levels of exosomal miR-92b-3p were significantly associated with poor cohesiveness (*p* = 0.0021), let-7g-5p and miR-146b-5p were significantly correlated with nerve infiltration (*p* = 0.0234 and *p* = 0.0126, respectively), and miR146b-5p was statistically correlated with tumor invasion depth in early-stage GC (*p* = 0.0089). In conclusion, serum exosomal miR-92b-3p, -146b-5p, -9-5p, and let-7g-5p may serve as potential non-invasive biomarkers for early diagnosis of GC.

## Introduction

Gastric cancer (GC) remains a leading cause of cancer-related mortality in the United States (Bray et al., 2018) and China (Chen et al., 2018). Because GC is mostly asymptomatic until it progresses to advanced stages, and lacking of an efficient biomarker with high sensitivity and specificity, the prognosis of GC patients remains poor. Hence, there is an urgent need to discover novel non-invasive biomarkers for the early diagnosis of GC.

Exosomes are 30–100 nm diameter, membrane-enclosed vesicles that are secreted by numerous cell types and present in various body fluids, such as blood, urine, saliva, etc. (Ohno et al., 2013). Exosomes act as mediators in intercellular communications by transporting proteins, lipids and nucleic acids to recipient cells, resulting in the modulation of different processes such as tumor invasion, angiogenesis, metastasis, and chemoresistance (Kosaka et al., 2016; Hu et al., 2019). The features of quick detection, convenient collection and minimal pain make exosomes serve as an ideal liquid biopsy tool for clinical application (Chi, 2016; Batth et al., 2017; Siravegna et al., 2017). MicroRNAs (miRNAs) are small non-coding RNAs (18–24 nt) that regulate protein translation. Extracellular miRNAs can be packaged into exosomes, which protects them from digestion by RNases (Manterola et al., 2014). High stability and enrichment of circulating exosomal miRNAs offer an attractive option for cancer diagnosis and prognosis. For example, serum exosomal miR-1246 and miR-21 could be used as promising diagnostic biomarkers for pancreatic cancer (Madhavan et al., 2015; Lai et al., 2017), prostate cancer (Bhagirath et al., 2018), and hepatocellular carcinoma (Sugimachi et al., 2015). To date, the majority of efforts are focused on breast cancer, prostate cancer, colorectal cancer, pancreatic cancer, hepatocellular carcinoma, and lung cancer (Jin et al., 2017). However, the relevance of serum exosomal miRNAs in early-stage GC has not been clearly elucidated.

We previously established an optimized procedure for screening and validating serum exosomal RNA biomarkers in prostate cancer and colorectal cancer (Huang et al., 2015; Wang et al., 2017). In this study, we used next-generation sequencing (NGS) to identify differential miRNA signatures in exosomes isolated from serum of early-stage GC patients, and provide a non-invasive method for early-stage GC detection. Finally, real-time qRT-PCR verified that the expression levels of exosomal miR-92b-3p, -146b-5p, -9-5p, and let-7g-5p were significantly higher in early-stage GC by comparison with matched healthy individuals, which established their utility as potential early-stage GC biomarkers.

## Materials and Methods

### Patients and Samples

**FIGURE 1:**
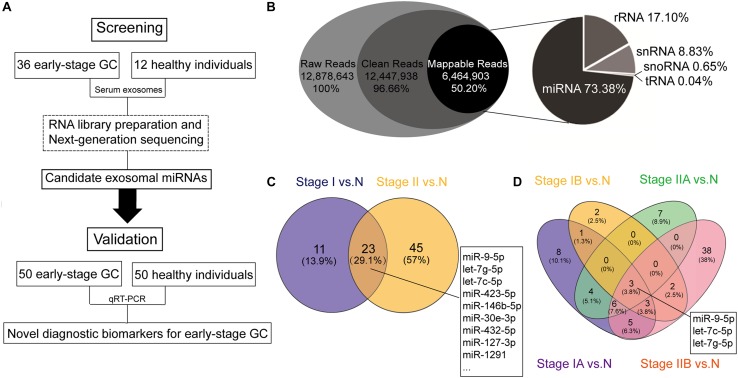
Schematic flowchart of the analytical pipeline and the results based on the next-generation sequencing (NGS). **(A)** Schematic flowchart of identification of early-stage gastric cancer (GC)-specific exosomal miRNAs. **(B)** RNA biotypes in the small RNA library prepared from human serum exosomes. Pie chart showing the mean percentage reads of serum exosomal small RNA library. Raw reads were the sequences obtained by RNA sequencing (RNA-seq). Clean reads were generated after read filtering and adapter trimming. Mappable reads were the RNA-seq reads mapped to known human RNAs, and were sorted into the following small ncRNAs: micro RNA (miRNA), ribosomal RNA (rRNA), small nuclear RNA (snRNA), small nucleolar RNA (snoRNA), and transfer RNA (tRNA). **(C)** Venn diagram showing overlap of the differentially expressed miRNAs between stage I gastric cancer vs. healthy individuals (stage I vs. N) and stage II gastric cancer vs. healthy individuals (stage II vs. N). **(D)** Venn diagram showing overlap of the differentially expressed miRNAs in all four stages (stages IA, IB, IIA, and IIB) vs. healthy individuals (N).

Serum samples were obtained from early-stage GC patients and healthy individuals at the Harbin Medical University Cancer Hospital between 2015 and 2016. The screening cohort included 36 early-stage GC patients, and 12 age and gender-matched healthy individuals. Fifty pairs of newly recruited early-stage GC patients and matched healthy individuals were included in the validation cohort. All serum samples were taken before treatment. All patients were histopathologically confirmed as gastric non-cardia adenocarcinoma patients (stages I and II) after operation. Clinical information including age, gender, pathological differentiation, pathological tumor stage, tumor invasion depth, presence of lymph node metastases, WHO cohesiveness, carcinoembryonic antigen (CEA) and carbohydrate antigen 199 (CA199) levels, and nerve infiltration were collected from medical records. The pathological tumor stage was identified according to the seventh edition AJCC TNM classification. CEA and CA199 levels were obtained from routine blood test before surgery, and CEA > 5 ng/ml and CA199 > 37 U/ml were defined as high levels. Inclusion criteria for healthy individuals, who were used as normal controls, included no malignancy, autoimmune disorders, endocrine disease, hepatitis, or HIV infection. The studies have been performed in accordance with the Declaration of Helsinki. All participants gave written informed consent, and the Harbin Medical University Cancer Hospital Ethics Committee approved the study. The overview flowchart of this study is shown in Figure 1A, and patient data are summarized in Table 1. Total 5 ml venous blood samples were collected before initial treatment, and was centrifugated at 3000 *g* for 10 min at 4°C within 2 h after collection. The supernatant (serum) was then transferred to RNase/DNase-free tubes and stored at −80°C until further processing.

**TABLE 1:** Clinicopathological features of early-stage gastric cancer patients in screening and validating cohort.

Variables	Screening cohort (%)	Validating cohort (%)	*p*-Value
Total number	36	50	
Age (mean ± SD)	56.50 ± 9.87	58.10 ± 10.41	0.557
**Gender**			
Female	11 (30.5)	12 (24.0)	0.622
Male	25 (69.5)	38 (76.0)	
**Pathological differentiation**			0.125
Poor	22 (61.1)	21 (42.0)	
Moderate + Well	14 (38.9)	29 (58.0)	
**Invasion depth**			0.986
T1	11 (30.5)	16 (32.0)	
T2	10 (27.8)	14 (28.0)	
T3	15 (41.7)	20 (40.0)	
**Lymph nodes metastasis**			0.374
Negative	29 (80.5)	44 (88.0)	
Positive	7 (19.5)	6 (12.0)	
**TNM stage**			0.782
IA	11 (30.5)	15 (30.0)	
IB	10 (27.8)	15 (30.0)	
IIA	8 (22.2)	14 (28.0)	
IIB	7 (19.5)	6 (12.0)	
**Poorly cohesive**			0.045
Negative	17 (47.2)	35 (70.0)	
Positive	19 (52.8)	15 (30.0)	
**Nerve infiltrate**			1
Negative	21 (58.3)	30 (60.0)	
Positive	15 (41.7)	20 (40.0)	
**CEA**			0.694
Normal	34 (94.4)	45 (90.0)	
High	2 (5.6)	5 (10.0)	
**CA199**			0.419
Normal	35 (97.2)	50 (100.0)	
High	1 (2.8)	0 (0.0)	

### Exosome Isolation

Exosomes were isolated using the ExoQuick exosome precipitation solution (System Biosciences, Mountain View, CA, United States) according to the manufacturer’s instructions with slight modifications. Briefly, 300 μl of cell-free serum samples were mixed with 75 μl of ExoQuick solution and RNase A (Sigma, St. Louis, MO, United States) to a final concentration of 10 μg/ml. The mixture was kept at 4°C overnight. Afterward, murine RNase inhibitor (NEB, Ipswich, MA, United States) was added to the mixture (150 units/ml) before centrifugation at 1500 *g* for 30 min. Finally, the supernatants were aspirated and the pelleted exosomes were re-suspended in 50 μl 1 × PBS, and immediately used for RNA extraction.

### Exosomal RNA Extraction and RNA Library Preparation

RNA of exosomes was isolated using miRNeasy micro kit (Qiagen, Valencia, CA, United States) according to the manufacturer’s protocol. The extracted RNA was eluted with 14 μl of RNase-free water. The library preparation was based on the protocols of Multiplex Small RNA Library Prep Set for Illumina (NEB, Ipswich, MA, United States) as previously described (Huang et al., 2013, 2015). Total 2 ng of isolated RNA was reverse-transcribed into cDNA sequencing libraries. Twelve sequencing libraries with different indices were pooled at a final amount of 2 nM and subjected to DNA sequencing.

### Sequencing Data Analysis

Next-generation sequencing was carried out on an Illumina HiSeq2000 platform by Novogen, Inc. (Beijing, China). The clustering of the index-coded samples was performed on a cBot Cluster Generation System using TruSeq SR Cluster kit v3-cBot-HS (Illumina). After cluster generation, the libraries were sequenced and 50 bp single-end reads were generated. Clean data were obtained by processing raw data in FASTQ format through custom perl and python scripts. Clean reads with certain range of length were mapped to reference sequence using Bowtie from miRBase (Release 20) and NCBI human genome reference sequences. The softwares miREvo and mirdeep2 were integrated to predict novel miRNA from the clean data. The DESeq R package (version 1.8.3) was used to identify differential expression of miRNAs between two groups. The *p*-values was adjusted using the Benjamini & Hochberg method. Corrected *p*-value of 0.05 was set as the threshold for significantly differential expression by default. To make the sequencing profiles comparable, we normalized RNA profiles as read count of a target RNA per million mapped reads (RPM).

### Real-Time Quantitative RT-PCR and Data Normalization

Some of the differentially expressed miRNAs identified by sequencing were validated using miScript SYBR Green PCR kit (Qiagen, Valencia, CA, United States). MiR-9-5p, let-7g-5p, let-7c-5p, miR-146b-5p, miR-101-3p, miR-92b-3p, miR-21-5p, and miR-26a-5p (MS00010752, MS00008337, MS00003129, MS00003542, MS00008372, MS00032144, MS00009079, MS00029239; Qiagen, Valencia, CA, United States) were selected. MiR-30e-5p (MS00009401; Qiagen, Valencia, CA, United States) was selected as the endogenous normalization control based on our previous study (Huang et al., 2015). Briefly, 4 ng of total serum exosomal RNA was reverse-transcribed into cDNA in a 20 μl reaction using the miScript II RT kit (Qiagen, Valencia, CA, United States), followed by dilution of the cDNA products for subsequent real-time PCR reactions. Then, the quantitative PCR was carried out on LightCycler^®^ 480 Real-Time PCR System (Roche Diagnostics, Mannheim, Germany) in a 384-well plate. The PCR reactions were set as follows: 95°C for 15 min, 40 cycles of 94°C for 15 s, 55°C for 30 s, and 72°C for 30 s. All quantitative experiments were independently repeated at least three times to remove any outliers.

### Data Processing and Statistical Analysis

Cycle threshold (Ct) values, obtained from real-time qPCR, were calculated as the expression data for miRNAs. ΔCt values were analyzed directly to compare different exosomal miRNA transcription levels (Silver et al., 2006). ΔCt = Ct_target miRNA_ – Ct_miR–__30__e–__5__*p*_, and lower ΔCt values indicated higher expression levels of exosomal miRNAs. Fold change (FC) was based on FC = 2^–ΔΔ*Ct*^, and represented as follows: if FC > 1, true FC = FC and if FC < 1, true FC = −1/FC. The subsequent statistical analyses were performed using GraphPad Prism version 6.0 (GraphPad Software, La Jolla, CA, United States) and SPSS software version 20.0 (IBM, Corp., Armonk, NY, United States). Data were presented as the mean ± SD. Unpaired Student’s *t*-test was used to analyze the differences in miRNA expression between early-stage GC patients and healthy individuals. Receiver operating characteristic (ROC) curve was used to evaluate the diagnostic power of the candidate exosomal miRNAs for early-stage GC. The cut-off value was determined using the Youden Index. Logistic regression adjusted for sex and age was utilized to establish the combination of exosomal miRNAs for GC diagnosis, so did the combined models based on exosomal miRNAs and traditional biomarkers. The performance of the combined models was evaluated by the area under the ROC curves (AUC). A two-sided *p* < 0.05 was considered statistically significant.

## Results

### Mapping of RNA Sequencing

The average raw reads from the RNA sequencing libraries were about 13 million, from which approximately 12.5 million (96.66%) reads with certain range of length (clean reads) were received after trimming. Among these, about 6.5 million reads (50.20%) were mapped to known RNA species. We annotated the mapped reads into the following biotypes of small ncRNAs: miRNAs, ribosomal RNAs (rRNAs), small nuclear RNAs (snRNAs), small nucleolar RNAs (snoRNAs), and transfer RNAs (tRNAs). Analysis of the mapped reads revealed miRNAs were the most common, accounting for 73.38% of all mappable RNAs, followed by 17.10% rRNAs, 8.83% snRNAs, 0.65% snoRNAs, and 0.04% tRNAs (Figure 1B). The raw sequencing data have been deposited in the Gene Expression Omnibus database (accession number: GSE130654).

### Exosomal MiRNA Profiling in the Screening Stage

Next, to identify candidate serum exosomal miRNAs for early-stage GC diagnosis, miRNAs with log2 transformed read counts < 5 and *p* > 0.05 were removed from the RNA sequencing data. A total of 79 known miRNAs remained after the initial screening step, including 66 up-regulated and 13 down-regulated miRNAs compared to normal control. Further systematic evaluation on the differential expression of the remaining miRNAs using Venn diagrams (Figure 1C) revealed that 23 miRNAs were significantly aberrant in both stage I and stage II GC when compared with normal control (Supplementary Table S1), 11 were uniquely found between stage I GC and normal control, and 45 miRNAs between stage II GC and normal control. When the early stage GC was further stratified into stages IA, IB, IIA, and IIB, miR-9-5p, let-7g-5p, and let-7c-5p were the only three miRNAs significantly aberrant in all early stages of GC, and miR-146b-5p was significantly aberrant in stages IA, IIA and IIB, but not in stage IB (Figure 1D). Meanwhile, many serum exosomal miRNAs were differentially expressed in a unique stage, suggesting the complexity of the miRNA transcriptome in tumorigenesis of GC. For example, miR-21-5p and miR-26-5p were significantly aberrant in stage IIB.

To assess the diagnostic value of the aberrantly expressed miRNAs, we performed ROC analysis based on the expression levels of the miRNAs. The miRNAs with *p* < 0.05, AUC ≥ 0.75, and both sensitivity and specificity at least ≥ 60% were selected as potential candidates for diagnostic markers. After screening, we selected eight serum exosomal miRNAs for downstream validation (Table 2).

**TABLE 2:** Diagnostic efficiencies of serum exosomal miRNA candidates in early-stage gastric cancer.

Column ID	AUC	*p*-Value	Cut-off	Sensitivity %	Specificity %
miR-9-5p	0.920	<0.0001	>11.36	80	90.91
let-7g-5p	0.850	0.0005	>11.42	74.29	90.91
let-7c-5p	0.886	0.0001	>12.03	80	90.91
miR-146b-5p	0.784	0.0048	>8.705	71.43	81.82
miR-92b-3p	0.788	0.0304	>13.04	66.67	90.91
miR-101-3p	0.805	0.0057	>10.28	75	90.91
miR-21-5p	1.000	0.0005	>12.35	100	90.91
miR-26a-5p	0.961	0.0013	>12.96	85.71	90.91

*AUC, area under the receiver operating characteristic curve.*

### Evaluation of the Diagnostic Potential of Exosomal MiRNAs for Early-Stage GC

**FIGURE 2:**
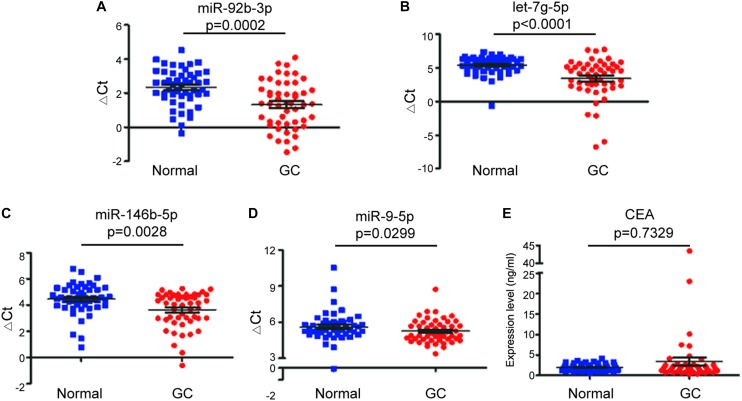
Comparison of expression of exosomal miRNAs in early-stage GC patients (GC) and healthy individuals (Normal). The scatter plots of the mean ± SD of ΔCt values of miR-92b-3p **(A)**, let-7g-5p **(B)**, miR-146b-5p **(C)**, and miR-9-5p **(D)**, and the mean ± SD of expression levels of serum CEA **(E)** between early-stage GC patients and healthy individuals.

**FIGURE 3:**
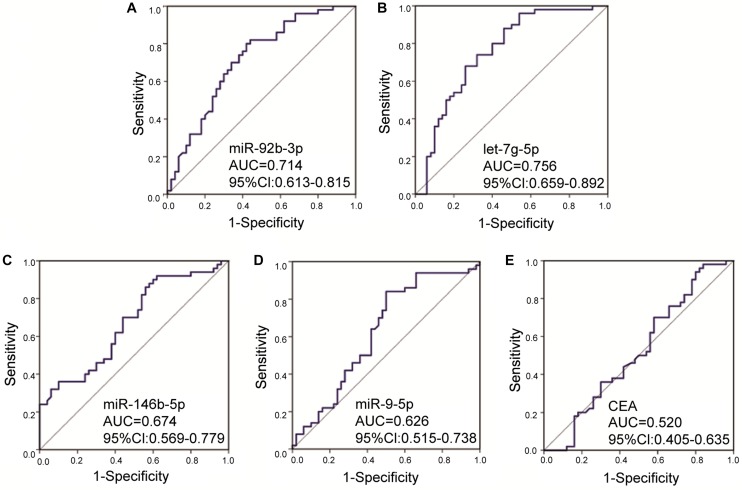
Receiver operating characteristic curves for the serum exosomal miRNAs and CEA to discriminate early-stage gastric cancer patients from healthy individuals. The AUC values of miR-92b-3p **(A)**, let-7g-5p **(B),** miR-146b-5p **(C)**, miR-9-5p **(D)**, and CEA **(E)** were 0.714 (*p* = 0.0002), 0.756 (*p* < 0.0001), 0.674 (*p* = 0.0028), 0.626 (*p* = 0.0299), and 0.520 (*p* = 0.7329), respectively. AUC, area under the receiver operating characteristic curve; CI, confidence interval.

To investigate whether candidate exosomal miRNAs in serum could be used as a potential biomarker, an independent patient cohort was validated by real-time qRT-PCR. Among the eight candidates, only four miRNAs were found to be potential diagnostic biomarkers for early-stage GC patients. As shown in Figures 2A–D, the ΔCt values of miR-92b-3p, let-7g-5p, miR-146b-5p, and miR-9-5p were significantly lower in early-stage GC patients as compared to healthy individuals (*p* < 0.05). Meanwhile, serum levels of CEA failed to distinguish early-stage GC patients from healthy individuals (*p* = 0.7329) (Figure 2E). Compared to healthy individuals, the expression levels of miR-92b-3p, let-7g-5p, miR-146b-5p, and miR-9-5p were 2.018, 3.926, 1.784, and 1.266-fold higher, respectively, in early-stage GC patients (Supplementary Table S2). We assessed the diagnostic capacity of each miRNA by computing their ROC curve. The results are illustrated in Figures 3A–D. Among the four candidates, let-7g-5p possessed the highest diagnostic power in discriminating early-stage GC patients and healthy individuals with AUC at 0.756 (95% CI, 0.659–0.892, cut-off < 4.184, sensitivity = 54%, specificity = 88%), followed by miR-92b-3p at 0.714 (95% CI, 0.613–0.815, cut-off < 1.690, sensitivity = 58%, specificity = 80%), miR-146b-5p at 0.674 (95% CI, 0.569–0.779, cut-off < 3.784, sensitivity = 46%, specificity = 82%), and miR-9-5p at 0.626 (95% CI, 0.515–0.738, cut-off < 4.932, sensitivity = 50%, specificity = 84%). In contrast, the AUC value of CEA was 0.520 (95% CI, 0.405–0.635, cut-off < 1.440, sensitivity = 38%, specificity = 70%) (Figure 3E).

### Combining Multiple Biomarkers Enhances the Diagnostic Power for Early-Stage GC

We next explored whether combination of the four exosomal miRNAs could be used to better discriminate early-stage GC patients from healthy individuals. As shown in Table 3, totally seven combined panels hold stronger diagnostic power than any single exosomal miRNA. The most powerful diagnostic panel consisting of miR-92b-3p and let-7g-5p received a AUC of 0.775, with sensitivity of 64% and specificity of 78%. The most sensitive diagnostic panel was the combination of miR-92b-3p + miR-146b-5p + miR-9-5p (68%), and the combination of miR-146b-5p + miR-9-5p was the most specific panel (88%). Moreover, significant synergistic effect was also found in the combinations of exosomal miRNA panels and traditional biomarkers (Table 3). The combined diagnosis of exosomal miR-92b-3p + let-7g-5p + miR-146b-5p + miR-9-5p with CEA had the most powerful efficiency with an AUC up to 0.786. Interestingly, compared with CEA and the exosomal miRNA panels, the combinations of CEA and any exosomal miRNA panel led to a mild loss of sensitivities but dramatically improved specificities. Among the different combined panels, the best specificity in the panel consisting of miR-92b-3p + miR-146b-5p + miR-9-5p + CEA reached 96% with an AUC of 0.758 (Table 3). Our results indicated that the exosomal miRNA combination panels and the combination of exosomal miRNAs with CEA could serve as potential predictive markers for the diagnosis of early-stage GC.

**TABLE 3:** ROC curve analysis on combinations of serum exosomal miRNAs for early-stage gastric cancer.

miRNA combination	AUC	*p*-Value	95% CI	Cut-off	Sensitivity %	Specificity %
miR-92b-3p + let-7g-5p	0.775	<0.0001	0.684–0.866	>0.484	64	78
miR-92b-3p + miR-146b-5p	0.736	<0.0001	0.638–0.834	>0.536	60	82
miR-146b-5p + miR-9-5p	0.705	0.0004	0.604–0.806	>0.607	44	88
miR-92b-3p + let-7g-5p + miR-146b-5p	0.774	<0.0001	0.683–0.865	>0.534	58	86
miR-92b-3p + let-7g-5p + miR-9-5p	0.774	<0.0001	0.684–0.865	>0.525	60	82
miR-92b-3p + miR-146b-5p + miR-9-5p	0.75	<0.0001	0.654–0.845	>0.492	68	74
miR-92b-3p + let-7g-5p + miR-146b-5p + miR-9-5p	0.773	<0.0001	0.682–0.864	>0.526	60	84
miR-92b-3p + CEA	0.722	0.0001	0.622–0.823	>0.527	60	80
miR-92b-3p + let-7g-5p + CEA	0.784	<0.0001	0.694–0.875	>0.599	58	90
miR-92b-3p + miR-146b-5p + CEA	0.743	<0.0001	0.645–0.841	>0.596	56	88
miR-92b-3p + miR-9-5p + CEA	0.724	0.0001	0.624–0.824	>0.533	58	80
miR-146b-5p + miR-9-5p + CEA	0.709	0.0003	0.609–0.810	>0.584	48	90
miR-92b-3p + let-7g-5p + miR-146b-5p + CEA	0.784	<0.0001	0.693–0.874	>0.640	54	94
miR-92b-3p + let-7g-5p + miR-9-5p + CEA	0.784	<0.0001	0.694–0.874	>0.599	58	90
miR-92b-3p + miR-146b-5p + miR-9-5p + CEA	0.758	<0.0001	0.663–0.853	>0.701	48	96
miR-92b-3p + let-7g-5p + miR-146b-5p + miR-9-5p + CEA	0.786	<0.0001	0.695–0.876	>0.613	58	90

*AUC, area under the receiver operating characteristic curve; CI, confidence interval; ROC, receiver operating characteristic.*

### Correlation of Serum Exosomal MiRNAs With Clinicopathological Features of Early-Stage GC

**FIGURE 4:**
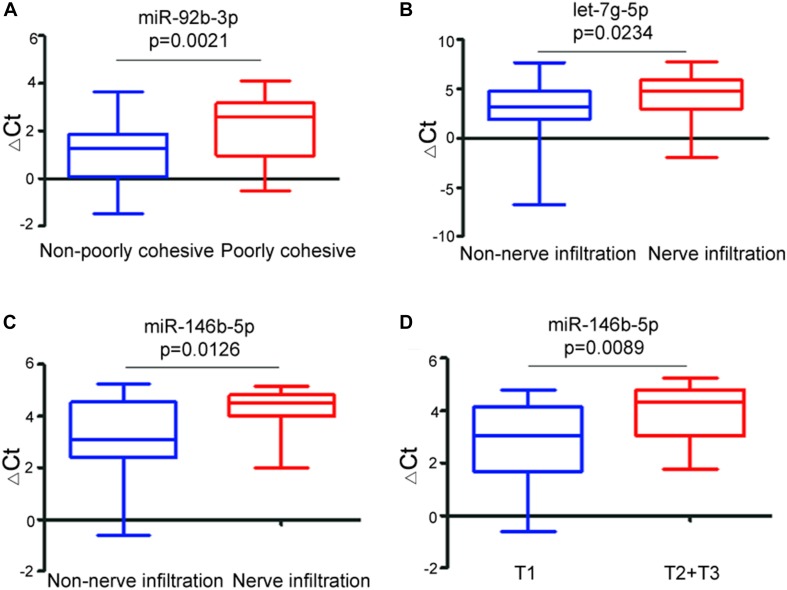
Relationship between the ΔCt values of exosomal miRNA biomarkers and clinicopathological features of early-stage gastric cancer. Boxplot showing differential ΔCt values of miR-92b-3p between non-poorly cohesive and poorly cohesive GC patients **(A)**, differential ΔCt values of let-7g-5p **(B)** and miR-146b-5p **(C)** between non-nerve infiltration and nerve infiltration gastric cancer patients, and differential ΔCt values of miR-146b-5p between T1 stage and T2 + T3 stage gastric cancer patients **(D)**.

Next, we focused on the relationship between the miRNAs and the clinicopathological features of early-stage GC patients. There was no statistical association between miR-92b-3p, let-7g-5p, miR-146b-5p, and miR-9-5p with age, gender, pathological differentiation, and lymph node metastases. However, the level of miR-92b-3p was significantly (*p* = 0.0021) lower in non/poorly cohesive GC patients (Figure 4A), the levels of let-7g-5p and miR-146b-5p were significantly (*p* = 0.0234 and *p* = 0.0126, respectively) lower in non/low nerve infiltration GC patients (Figures 4B,C), and the level of miR146b-5p was significantly (*p* = 0.0089) increased with the increase in tumor invasion depth (Figure 4D). In this dataset, we did not find any statistical correlations between miR-9-5p and nerve infiltration, poorly cohesive, and invasion depth (*p* > 0.05).

### Expression of MiRNAs in GC Tissues

To further clarify the relationship between the identified exosomal miRNA biomarkers and GC, the miRNA expression data derived from 446 GC tissues and 45 normal gastric tissues were downloaded from The Cancer Genome Atlas (TCGA) database.^1^ We used DESeq R package (version 1.8.3) to identify differentially expressed miRNAs between the two groups. Compared to normal gastric tissues, the expression levels of miR-92b-3p and miR-146b-5p were significantly upregulated in GC tissues (*p* = 0.0042 and *p* < 0.0001, respectively), and the expression levels of let-7g-5p and miR-9-5p were significantly downregulated in GC tissues (*p* = 0.0003 and *p* = 0.0026, respectively) (Supplementary Figure S1).

## Discussion

Although treatments for GC have advanced in recent decades, the early diagnosis relies on traditional imaging examinations, biopsy, or serum biomarkers. Due to inherent clinical heterogeneity, early diagnosis continues to be one of the key challenges in GC. As we known, reliable molecular markers play vital roles in detecting cancers, monitoring recurrence, assessing prognosis, and improving effective personalized cancer therapy (Zhou et al., 2018; Sun et al., 2019). Thus, development of a new non-invasive diagnostic method has become critical to improve therapeutic effect. In this study, we demonstrated that a panel of the circulating exosomal miRNAs had higher diagnostic efficacies than traditional biomarker CEA in early-stage GC. Meanwhile, synergistic effects were also found in combining CEA with the exosomal miRNA markers. These results provided evidence for exosomal miRNAs as a new non-invasive diagnostic option for early-stage GC.

Emerging evidence suggests that exosomal miRNAs can serve as potential biomarkers of GC, and are eligible for diagnosis, predicting recurrences, and providing prognostic information. Serum exosomal miR-10b-5p, miR-195-5p, miR-20a-3p, miR-296-5p were identified as novel potential biomarkers for detecting GC (Huang et al., 2017b). Low levels of plasma exosomal miRNA-101 and miR-23b have been confirmed to be related to poor prognosis in GC patients, and plasma exosomal miR-23b can be used as a minimally invasive predictive biomarker for the recurrence of GC (Kumata et al., 2018). Additionally, exosomal miR-21 and miR-1225-5p derived from peritoneal lavage fluid could be novel biomarkers for peritoneal dissemination after curative resection of GC (Tokuhisa et al., 2015). Even though promising advances have been made in using exosomal miRNAs as diagnostic biomarkers for GC in recent years, the GCs involved in the previous studies are composed of both early and advanced stages (stage I to stage IV). Early diagnosis of GC remains a challenge and non-invasive evidence on diagnosis of early-stage GC is lacking. Hence, reliable biomarkers with high sensitivity and specificity need to be developed. To the best of our knowledge, this is the first study involved only early-stage patients (stage I and stage II) to analysis the early diagnostic value of serum exosomal miRNAs in GC.

MiR-92b-3p has been confirmed to play an important role in cancer proliferation, invasion, and migration. As an oncogenic miRNA, miR-92b-3p promotes carcinogenesis and metastasis by down-regulating F-box and WD-40 domain protein 7 (FBXW7/hCdc4) in colorectal cancer (Gong et al., 2018). In accordance with this evidence, we found that up-regulated miR-92b-3p was associated with early-stage GC. Besides, let-7g-5p has been investigated in melanoma, lung adenocarcinoma, ovarian cancer, glioblastoma, and renal cell carcinoma (RCC) (Gowrishankar et al., 2014; Petrillo et al., 2016). Overexpression of let-7g-5p significantly inhibits cell proliferation and migration, and could be a biomarker of post-operative recurrence and prognosis (Petrillo et al., 2016). Let-7g-5p was clustered into the let-7g family. Down-regulated let-7g has been associated with poor survival and lymph node metastasis in GC (Ueda et al., 2010). However, the role of let-7g-5p in GC had not been explored. This is the first study to confirm that aberrant expression of serum exosomal let-7g-5p was associated with early-stage GC. Consistent with our data, miR-146b-5p was observed to be up-regulated in colorectal cancer and renal cell carcinoma (Zhu et al., 2017), but frequently down-regulated in pancreatic cancer and glioblastoma (Li et al., 2013). Recent studies showed that deregulation of miR-146b-5p in GC tissue was associated with the tumor stage, and could be used as a prognosis biomarker (Zhou et al., 2013). In this study, we further verified the diagnostic role of serum exosomal miR-146b-5p in early-stage GC. Moreover, we found that serum exosomal miR-9-5p was up-regulated in early-stage GC. Likewise, up-regulation of miR-9-5p was observed in both breast cancer and lung cancer, which promoted tumor proliferation, invasion, and metastasis (Barbano et al., 2017). However, functional roles of miR-146b-5p and miR-9-5p in GC remain unknown.

It is of note that the molecular profile of tumors dynamically changes among patients as well as over time. In terms of miRNA profile, the expression signatures are also significantly varies as the medical condition of the specific donor differs. Among the biomarkers for early diagnosis of GC, the levels of miR-92b in plasma and miR-146b-5p in tissue have also been found significantly different between early- and late-stage GC (Chen et al., 2013; Zhou et al., 2015). Additionally, differential expression of let-7g-5p in tissue has been found between early- and late-stage clear cell renal carcinoma (Gowrishankar et al., 2014). However, the evidences for the differential expression of miR-9-5p and let-7g-5p between early- and late-stage GC are still lacking. These phenomena suggest the exosomal miRNA biomarkers for early diagnosis of cancer are not necessarily aberrantly expressed throughout the process of GC.

It has been reported that different expression levels of miRNAs could be frequently observed between circulating and tissue samples (Valadi et al., 2007; Huang et al., 2017a). In our study, based on TCGA data, we found that miR-92b-3p and miR-146b-5p were significantly upregulated in GC tissues, while let-7g-5p and miR-9-5p were significantly downregulated in GC tissues. We suspected that the discrepancies of the expression signatures between tissues and serum exosomes might be due to the following explanations. First, the absorption and degradation efficiency of circulating exosomal miRNAs and tissue miRNAs are quite different, and varieties in techniques and sequencing platform might also contribute to the discrepancies (Rabinowits et al., 2017). Second, compared with circulating exosomal miRNA reflecting the systematic disease status of GC, tissue miRNAs just presented the landscape of local changes. Currently, although significant advances have been achieved in exosome study, the understanding of the packaging and releasing mechanism of circulating exosomal miRNAs is still far from complete. Intensive work is still required to clarify the relationship of miRNAs between circulating exosomes and tissues.

In this study, combination of serum CEA and a panel of serum exosomal miRNAs (miR-92b-3p + let-7g-5p + miR-146b-5p + miR-9-5p) reached the highest diagnostic power for early stage GC, which could relieve a substantial proportion of patients from invasive biopsy. Nonetheless, this study had several potential limitations. First, although the results successfully showed four GC-related exosomal miRNAs, the number of early-stage GC patients enrolled in the study was limited. Further studies are necessary to evaluate these exosomal miRNA signatures in a larger cohort of early-stage GC patients. Second, the heterogeneous patient population could impact overall accuracy of the exosomal miRNAs, and our future study will compare the exosomal miRNAs with diverse tumor types. Third, we cannot elucidate the origin of the diagnostic miRNA markers. Whether they are derived from GC cells requires further investigation.

## Conclusion

This study demonstrated the diagnostic values of serum exosomal miR-92b-3p, -146b-5p, -9-5p, and let-7g-5p in early-stage GC, and suggested the possibility of non-invasive circulating exosomal miRNAs as an alternative to conventional invasive approaches in detection of GC in the future. Moreover, large-scale validation is still required to confirm the potential applicability of these markers in GC diagnosis.

## Data Availability Statement

The raw sequencing data for this study can be found in the Gene Expression Omnibus database (accession number: GSE130654).

## Ethics Statement

The studies involving human participants were reviewed and approved by the Harbin Medical University Cancer Hospital Ethics Committee. The patients/participants provided their written informed consent to participate in this study.

## Author Contributions

ST, YZ, and XH conceived and designed the study. ST and JC performed the experiments. YY and CL collected the samples and organized the clinical information. LW was responsible for the data processing and analysis of next-generation sequencing. ST performed statistical analysis and wrote the manuscript. YY, YZ, and XH reviewed and edited the manuscript. All the authors read and approved the final manuscript.

## Conflict of Interest

The authors declare that the research was conducted in the absence of any commercial or financial relationships that could be construed as a potential conflict of interest.

## Footnotes

1 cancergenome.nih.gov/

## References

[B1] Barbano R. Pasculli B. Rendina M. Fontana A. Fusilli C. Copetti M. (2017). Stepwise analysis of MIR9 loci identifies miR-9-5p to be involved in Oestrogen regulated pathways in breast cancer patients. *Sci. Rep.* 7:45283. 10.1038/srep45283 28345661 PMC5366901

[B2] Batth I. S. Mitra A. Manier S. Ghobrial I. M. Menter D. Kopetz S. (2017). Circulating tumor markers: harmonizing the yin and yang of CTCs and ctDNA for precision medicine. *Ann. Oncol.* 28 468–477. 10.1093/annonc/mdw619 27998963 PMC6246190

[B3] Bhagirath D. Yang T. L. Bucay N. Sekhon K. Majid S. Shahryari V. (2018). microRNA-1246 is an exosomal biomarker for aggressive prostate cancer. *Cancer Res.* 78 1833–1844. 10.1158/0008-5472.CAN-17-2069 29437039 PMC5890910

[B4] Bray F. Ferlay J. Soerjomataram I. Siegel R. L. Torre L. A. Jemal A. (2018). Global cancer statistics 2018: GLOBOCAN estimates of incidence and mortality worldwide for 36 cancers in 185 countries. *CA Cancer J. Clin.* 68 394–424. 10.3322/caac.21492 30207593

[B5] Chen W. Sun K. Zheng R. Zeng H. Zhang S. Xia C. (2018). Cancer incidence and mortality in China, 2014. *Chin. J. Cancer Res.* 30 1–12. 10.21147/j.issn.1000-9604.2018.01.01 29545714 PMC5842223

[B6] Chen Z. Saad R. Jia P. Peng D. Zhu S. Washington M. K. (2013). Gastric adenocarcinoma has a unique microRNA signature not present in esophageal adenocarcinoma. *Cancer* 119 1985–1993. 10.1002/cncr.28002 23456798 PMC3731210

[B7] Chi K. R. (2016). The tumour trail left in blood. *Nature* 532 269–271. 10.1038/532269a 27075102

[B8] Gong L. Ren M. Lv Z. Yang Y. Wang Z. (2018). miR-92b-3p promotes colorectal carcinoma cell proliferation, invasion, and migration by inhibiting FBXW7 *in vitro* and *in vivo*. *DNA Cell Biol.* 37 501–511. 10.1089/dna.2017.4080 29638162

[B9] Gowrishankar B. Ibragimova I. Zhou Y. Slifker M. J. Devarajan K. Al-Saleem T. (2014). MicroRNA expression signatures of stage, grade, and progression in clear cell RCC. *Cancer Biol. Ther.* 15 329–341. 10.4161/cbt.27314 24351440 PMC3974834

[B10] Hu Y. Qi C. Liu X. Zhang C. Gao J. Wu Y. (2019). Malignant ascites-derived exosomes promote peritoneal tumor cell dissemination and reveal a distinct miRNA signature in advanced gastric cancer. *Cancer Lett.* 457 142–150. 10.1016/j.canlet.2019.04.034 31075288

[B11] Huang X. Yuan T. Liang M. Du M. Xia S. Dittmar R. (2015). Exosomal miR-1290 and miR-375 as prognostic markers in castration-resistant prostate cancer. *Eur. Urol.* 67 33–41. 10.1016/j.eururo.2014.07.035 25129854 PMC4252606

[B12] Huang X. Yuan T. Tschannen M. Sun Z. Jacob H. Du M. (2013). Characterization of human plasma-derived exosomal RNAs by deep sequencing. *BMC Genomics* 14:319. 10.1186/1471-2164-14-319 23663360 PMC3653748

[B13] Huang Z. Zhang L. Zhu D. Shan X. Zhou X. Qi L. W. (2017a). A novel serum microRNA signature to screen esophageal squamous cell carcinoma. *Cancer Med.* 6 109–119. 10.1002/cam4.973 28035762 PMC5269712

[B14] Huang Z. Zhu D. Wu L. He M. Zhou X. Zhang L. (2017b). Six serum-based miRNAs as potential diagnostic biomarkers for gastric cancer. *Cancer Epidemiol. Biomarkers Prev.* 26 188–196. 10.1158/1055-9965.EPI-16-0607 27756776

[B15] Jin X. Chen Y. Chen H. Fei S. Chen D. Cai X. (2017). Evaluation of tumor-derived exosomal miRNA as potential diagnostic biomarkers for early-stage non-small cell lung cancer using next-generation sequencing. *Clin. Cancer Res.* 23 5311–5319. 10.1158/1078-0432.CCR-17-0577 28606918

[B16] Kosaka N. Yoshioka Y. Fujita Y. Ochiya T. (2016). Versatile roles of extracellular vesicles in cancer. *J. Clin. Invest.* 126 1163–1172. 10.1172/JCI81130 26974161 PMC4811151

[B17] Kumata Y. Iinuma H. Suzuki Y. Tsukahara D. Midorikawa H. Igarashi Y. (2018). Exosomeencapsulated microRNA23b as a minimally invasive liquid biomarker for the prediction of recurrence and prognosis of gastric cancer patients in each tumor stage. *Oncol. Rep.* 40 319–330. 10.3892/or.2018.6418 29749537

[B18] Lai X. Wang M. McElyea S. D. Sherman S. House M. Korc M. (2017). A microRNA signature in circulating exosomes is superior to exosomal glypican-1 levels for diagnosing pancreatic cancer. *Cancer Lett.* 393 86–93. 10.1016/j.canlet.2017.02.019 28232049 PMC5386003

[B19] Li Y. Wang Y. Yu L. Sun C. Cheng D. Yu S. (2013). miR-146b-5p inhibits glioma migration and invasion by targeting MMP16. *Cancer Lett.* 339 260–269. 10.1016/j.canlet.2013.06.018 23796692

[B20] Madhavan B. Yue S. Galli U. Rana S. Gross W. Muller M. (2015). Combined evaluation of a panel of protein and miRNA serum-exosome biomarkers for pancreatic cancer diagnosis increases sensitivity and specificity. *Int. J. Cancer* 136 2616–2627. 10.1002/ijc.29324 25388097

[B21] Manterola L. Guruceaga E. Gallego Perez-Larraya J. Gonzalez-Huarriz M. Jauregui P. Tejada S. (2014). A small noncoding RNA signature found in exosomes of GBM patient serum as a diagnostic tool. *Neuro Oncol.* 16 520–527. 10.1093/neuonc/not218 24435880 PMC3956347

[B22] Ohno S. Ishikawa A. Kuroda M. (2013). Roles of exosomes and microvesicles in disease pathogenesis. *Adv. Drug Deliv. Rev.* 65 398–401. 10.1016/j.addr.2012.07.019 22981801

[B23] Petrillo M. Zannoni G. F. Beltrame L. Martinelli E. DiFeo A. Paracchini L. (2016). Identification of high-grade serous ovarian cancer miRNA species associated with survival and drug response in patients receiving neoadjuvant chemotherapy: a retrospective longitudinal analysis using matched tumor biopsies. *Ann. Oncol.* 27 625–634. 10.1093/annonc/mdw007 26782955

[B24] Rabinowits G. Bowden M. Flores L. M. Verselis S. Vergara V. Jo V. Y. (2017). Comparative analysis of MicroRNA expression among benign and malignant tongue tissue and plasma of patients with tongue cancer. *Front. Oncol.* 7:191. 10.3389/fonc.2017.00191 28900608 PMC5581802

[B25] Silver N. Best S. Jiang J. Thein S. L. (2006). Selection of housekeeping genes for gene expression studies in human reticulocytes using real-time PCR. *BMC Mol. Biol.* 7:33. 10.1186/1471-2199-7-33 17026756 PMC1609175

[B26] Siravegna G. Marsoni S. Siena S. Bardelli A. (2017). Integrating liquid biopsies into the management of cancer. *Nat. Rev. Clin. Oncol.* 14 531–548. 10.1038/nrclinonc.2017.14 28252003

[B27] Sugimachi K. Matsumura T. Hirata H. Uchi R. Ueda M. Ueo H. (2015). Identification of a bona fide microRNA biomarker in serum exosomes that predicts hepatocellular carcinoma recurrence after liver transplantation. *Br. J. Cancer* 112 532–538. 10.1038/bjc.2014.621 25584485 PMC4453648

[B28] Sun J. Zhao H. Lin S. Bao S. Zhang Y. Su J. (2019). Integrative analysis from multi-centre studies identifies a function-derived personalized multi-gene signature of outcome in colorectal cancer. *J. Cell Mol. Med.* 23 5270–5281. 10.1111/jcmm.14403 31140730 PMC6653159

[B29] Tokuhisa M. Ichikawa Y. Kosaka N. Ochiya T. Yashiro M. Hirakawa K. (2015). Exosomal miRNAs from peritoneum lavage fluid as potential prognostic biomarkers of peritoneal metastasis in gastric cancer. *PLoS One* 10:e0130472. 10.1371/journal.pone.0130472 26208314 PMC4514651

[B30] Ueda T. Volinia S. Okumura H. Shimizu M. Taccioli C. Rossi S. (2010). Relation between microRNA expression and progression and prognosis of gastric cancer: a microRNA expression analysis. *Lancet Oncol.* 11 136–146. 10.1016/S1470-2045(09)70343-2 20022810 PMC4299826

[B31] Valadi H. Ekstrom K. Bossios A. Sjostrand M. Lee J. J. Lotvall J. O. (2007). Exosome-mediated transfer of mRNAs and microRNAs is a novel mechanism of genetic exchange between cells. *Nat. Cell Biol.* 9 654–659. 10.1038/ncb1596 17486113

[B32] Wang J. Yan F. Zhao Q. Zhan F. Wang R. Wang L. (2017). Circulating exosomal miR-125a-3p as a novel biomarker for early-stage colon cancer. *Sci. Rep.* 7:4150. 10.1038/s41598-017-04386-1 28646161 PMC5482839

[B33] Zhou L. Zhao X. Han Y. Lu Y. Shang Y. Liu C. (2013). Regulation of UHRF1 by miR-146a/b modulates gastric cancer invasion and metastasis. *FASEB J.* 27 4929–4939. 10.1096/fj.13-233387 23982143

[B34] Zhou M. Hu L. Zhang Z. Wu N. Sun J. Su J. (2018). Recurrence-associated long non-coding RNA signature for determining the risk of recurrence in patients with colon cancer. *Mol. Ther. Nucleic Acids* 12 518–529. 10.1016/j.omtn.2018.06.007 30195788 PMC6076224

[B35] Zhou X. Zhu W. Li H. Wen W. Cheng W. Wang F. (2015). Diagnostic value of a plasma microRNA signature in gastric cancer: a microRNA expression analysis. *Sci. Rep.* 5:11251. 10.1038/srep11251 26059512 PMC4462022

[B36] Zhu Y. Wu G. Yan W. Zhan H. Sun P. (2017). miR-146b-5p regulates cell growth, invasion, and metabolism by targeting PDHB in colorectal cancer. *Am. J. Cancer Res.* 7 1136–1150. 28560062 PMC5446479

