# Supplementary material for: Combination of Four Serum Exosomal MiRNAs as Novel Diagnostic Biomarkers for Early-Stage Gastric Cancer
Source: Front Genet. 2020 Mar 17;11:237. doi: 10.3389/fgene.2020.00237 (PMC7089961; doi:10.3389/fgene.2020.00237)
Supplement: Supplementary file 1 [file Data_Sheet_1.docx]

Supplementary Material

**Supplementary Table S1**. Early-stage gastric cancer-related serum exosomal miRNAs (stage I and stage II compared with normal controls).

| **Column ID** | ***P* value** | **Mean(GC)** | **Mean(N)** | **MeanRatio**  **(GC/N)** | **Fold Change**  **(GC/N)** | **Description^*^** |
| --- | --- | --- | --- | --- | --- | --- |
| hsa-miR-3615 | 0.0268 | 10.639 | 10.990 | 0.784 | -1.277 | Down |
| hsa-miR-3184-3p | 0.0161 | 16.469 | 16.796 | 0.799 | -1.256 | Down |
| hsa-miR-151a-3p | 0.0179 | 11.805 | 12.124 | 0.801 | -1.248 | Down |
| hsa-miR-423-5p | 0.0190 | 16.527 | 16.796 | 0.830 | -1.204 | Down |
| hsa-let-7b-5p | 0.0255 | 15.144 | 14.807 | 1.264 | 1.264 | Up |
| hsa-miR-873-3p | 0.0373 | 8.5890 | 8.2412 | 1.273 | 1.273 | Up |
| hsa-miR-27b-3p | 0.0237 | 10.006 | 9.589 | 1.335 | 1.335 | Up |
| hsa-miR-139-5p | 0.0213 | 10.007 | 9.568 | 1.357 | 1.357 | Up |
| hsa-miR-432-5p | 0.0212 | 9.399 | 8.929 | 1.385 | 1.385 | Up |
| hsa-miR-127-3p | 0.0124 | 10.868 | 10.391 | 1.394 | 1.394 | Up |
| hsa-miR-221-3p | 0.0203 | 8.098 | 7.504 | 1.512 | 1.512 | Up |
| hsa-let-7a-5p | 0.0213 | 14.465 | 13.867 | 1.514 | 1.514 | Up |
| hsa-let-7c-5p | 0.0001 | 12.430 | 11.730 | 1.625 | 1.625 | Up |
| hsa-miR-7641 | 0.0292 | 6.180 | 5.450 | 1.661 | 1.661 | Up |
| hsa-let-7f-5p | 0.0095 | 13.815 | 13.076 | 1.674 | 1.674 | Up |
| hsa-miR-30e-3p | 0.0148 | 8.599 | 7.846 | 1.689 | 1.688 | Up |
| hsa-miR-146b-5p | 0.0048 | 9.073 | 8.300 | 1.715 | 1.715 | Up |
| hsa-let-7i-5p | 0.0084 | 14.608 | 13.838 | 1.725 | 1.725 | Up |
| hsa-let-7g-5p | 0.0005 | 11.791 | 11.004 | 1.735 | 1.735 | Up |
| hsa-miR-98-5p | 0.0098 | 8.438 | 7.505 | 1.940 | 1.940 | Up |
| hsa-miR-9-5p | < 0.0001 | 11.838 | 10.804 | 2.050 | 2.050 | Up |
| hsa-miR-1291 | 0.0196 | 5.225 | 3.409 | 3.523 | 3.523 | Up |
| hsa-miR-138-5p | 0.0154 | 5.444 | 2.866 | 5.973 | 5.973 | Up |

GC, gastric cancer; N, normal control. * The expression levels of miRNAs were calculated by comparing early-stage gastric cancer patients to normal controls.

**Supplementary Table S2**. Comparison of serum exosomal miR-92b-3p, let-7g-5p, miR-146b-5p and miR-9-5p between the early-stage gastric cancer patients and the controls.

| Δ**Ct of miRNA** | **Controls (mean ± SD)** | **Cases (mean ± SD)** | ***P*value** | **Fold change** |
| --- | --- | --- | --- | --- |
| miR-92b-3p | 2.334 ± 1.062 | 1.321 ± 1.380 | 0.0002 | ↑ 2.018 |
| Let-7g-5p | 5.409 ± 1.322 | 3.436 ± 2.966 | < 0.0001 | ↑ 3.926 |
| miR-146b-5p | 4.466 ± 1.136 | 3.631 ± 1.339 | 0.0028 | ↑ 1.784 |
| miR-9-5p | 5.600 ± 1.360 | 5.260 ± 0.967 | 0.0299 | ↑ 1.266 |

**Figure Legends**

**Supplementary Figure S1.** Expression of the four miRNAs in tumor tissues of gastric cancer based on TCGA data. Boxplot diagram showing differential expression of miR-92b-3p (A), miR-146b-5p (B), let-7g-5p (C), and miR-9-5p (D) in gastric cancer tissues and normal gastric tissues.

**
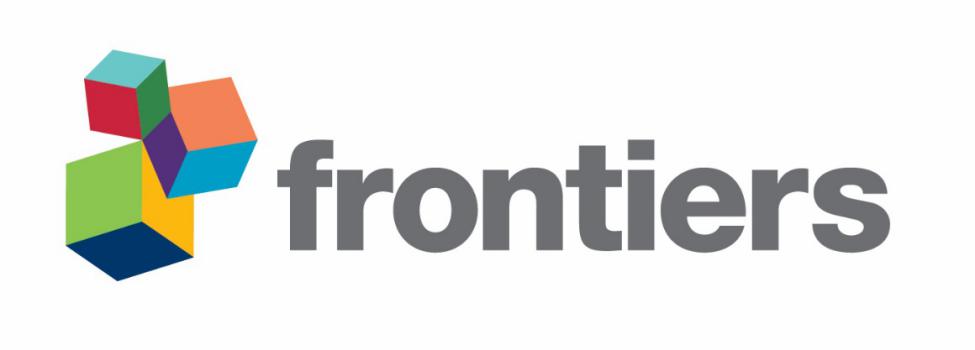
**
