# Supplementary figures and images for: Combination of Four Serum Exosomal MiRNAs as Novel Diagnostic Biomarkers for Early-Stage Gastric Cancer
Source: Front Genet. 2020 Mar 17;11:237. doi: 10.3389/fgene.2020.00237 (PMC7089961; doi:10.3389/fgene.2020.00237)

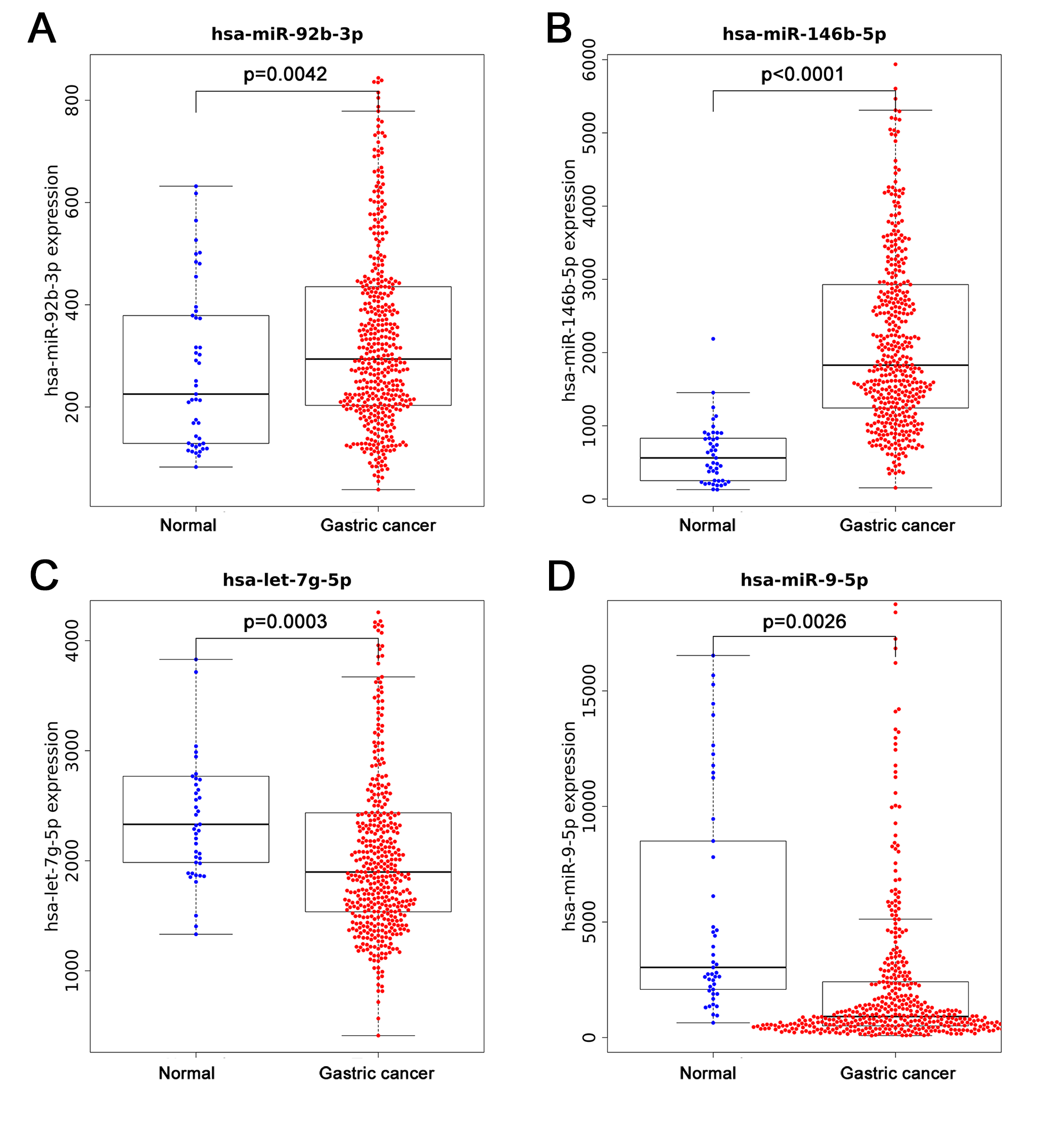

Supplement: Supplementary file 2 [file Image_1.TIF]
